# Canine decontamination, a laboratory study evaluating proper techniques to remove toxic materials from working dogs

**DOI:** 10.3389/fvets.2025.1649673

**Published:** 2025-09-26

**Authors:** Brian France, Thomas A. Malloy, Robert G. Buntz, Kelly A. Mann

**Affiliations:** ^1^TDA Research, Inc., Golden, CO, United States; ^2^Battelle Memorial Institute, Columbus, OH, United States; ^3^Mantel Technologies, Inc., Fort Collins, CO, United States; ^4^Department of Small Animal Primary Care, Midwestern University, Glendale, AZ, United States; ^5^Department of Clinical Sciences, Colorado State University, Fort Collins, CO, United States

**Keywords:** canine, military working dog, multi-purpose canine, chemical warfare agent, decontamination, sulfur mustard, venomous agent X

## Abstract

**Introduction:**

Working dogs can inadvertently encounter toxic chemicals while performing their key activities. These can include toxic industrial chemicals and materials (TICs/TIMs), pharmaceuticals, illicit drugs, sewage, pesticides, and even highly toxic chemical warfare agents. All these materials can poison the canine, be spread by touch, and can be transferred to the handler, vehicle, or veterinary medical staff. A successful decontamination technique must be safe for the handler to perform, can be performed at the site of contamination, successfully removes the hazardous material before it poisons the canine or transfers to other surfaces, and does not lead to a large hazardous waste disposal event.

**Materials:**

Canine cadaver tissue samples (intact skin/fur) were used to conduct a decontamination comparison between dry, waterless, wipe decontamination and traditional soap and water wash decontamination. The chemical warfare agents sulfur mustard (HD) and venomous agent X (VX) were used for all testing.

**Results:**

The dry, waterless, wipe decontamination removed more chemical toxin (HD and VX) from canine fur, preventing transfer to the skin. The soap and water wash decontamination provided a route of transfer for toxins to reach the canine skin.

**Discussion:**

To successfully decontaminate a working dog after toxic chemical exposure to HD and VX, dry, waterless, wipe decontamination should be performed to remove the majority of the toxin. This procedure reduces the transfer hazard to the handler, vehicle and veterinary medical staff which can then perform further decontamination and medical intervention.

## 1 Introduction

Protecting working dogs from exposure to hazardous chemicals and materials is especially challenging because protective equipment in the form of inhalation masks and suits essentially block the canine’s ability to perform key functions like detection, tracking, patrol and biting. Working dogs are at risk of exposure to a range of hazardous chemicals encountered during service. For traditional military and law enforcement, toxic chemicals and threat agents include nerve agents, vesicants (blister agents), incapacitating (BZ-type) agents, cyanide compounds (blood agents), choking agents, riot-control (irritating) agents, incendiary agents, and smoke agents. Working dogs can also contact toxic industrial chemicals (TIC) and toxic industrial materials (TIM) in an urban environment, after natural disasters or during urban search and rescue missions. Common types of industrial TICs and TIMs include hydrocarbons, polychlorinated biphenyls, hazardous metals, asbestos, soap and detergents, acids and bases, glycols, phenols, alcohols, harmful gasses, and hydrogen sulfide.

When a canine is exposed to toxic agents, their fur acts as the first line of defense against these exposures. Minimally haired and hairless areas like the paws, face and abdomen allow for rapid exposure to potentially toxic agents. Materials and chemicals that contact the fur must penetrate through the haircoat before skin exposure. Compounds that can wick through fur could result in rapid skin absorption. For non-wicking compounds, the fur can provide increased protection time before the skin becomes exposed. On the other hand, fur can also trap these contaminants, preventing easy removal (decontamination), and prolonging exposure while allowing for the possible transfer of contaminants to people, equipment, and otherwise uncontaminated facilities. Research studies for the decontamination of human skin have primarily focused on two areas: (1) identifying products that can decontaminate human skin (1) and (2) research that focuses on the concept of how decontamination should be performed (2). While research is ongoing in these areas of human decontamination, canine decontaminant products and decontamination methods derived from that research neglect the differences between human hair and skin and canine skin and fur.

The U.S. Army has published Field Manual 4–02.18 which provides guidelines on military working dog^1^ (MWD) protection from hazardous substances, including some recommendations on decontaminants and methods to decontaminate. The decontaminants recommended included Reactive Skin Decontamination Lotion (RSDL) for nerve and vesicant chemical warfare agents (CWA). RSDL is a U.S. Food and Drug Administration (FDA) registered medical device for human skin decontamination which neutralizes many CWAs. While RSDL has been approved for human skin decontamination, direct transfer to canine use is problematic. No published studies have been performed on the efficacy or safety of its use on canine fur or skin. Recent studies conducted by the U.S. Department of Health and Human Services have shown that the active ingredient in RSDL [Dekon 139 and 2,3-Butanedione monoxime (DAM)] has adverse effects in humans and has been shown to cause serious systemic toxicity issues (3). Further decontaminant guidelines from FM 4–02.18 only recommend the use of large amounts of soap and water. These recommendations are ambiguous at best, as no soap product or concentration of the solution is provided. In addition, the use of large amounts of soap and water are extremely logistically challenging and are not available quickly or in an austere environment, at the point of contamination.

**Figure 1:**
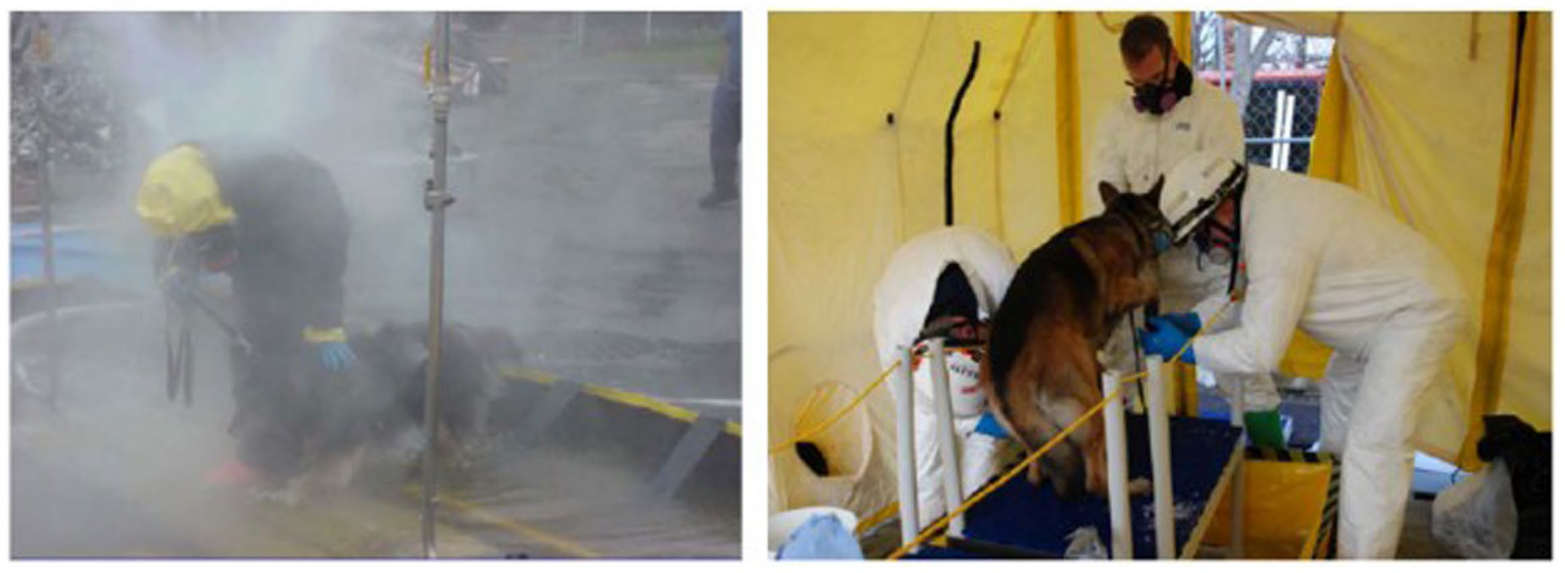
Canine in human gross decontamination system (left) and K9 Decon system during a field test (right); (4).

Concerned handlers and veterinarians have been left to identify products and procedures to perform these decontamination tasks. Dr. Lori Gordon, Veterinary Specialist from the Massachusetts Task Force 1 Urban Search and Rescue Response Team has published several reports and presentations on guidelines for emergency, gross, and technical decontamination of working canines (4). Dr. Gordon’s work began with human gross decontamination systems and methods and improved the techniques and equipment for a K9 Decon System (Figure 1). While large-scale canine decontamination procedures address many issues, there are several areas where significant improvements are required for working dogs. First, human and canine wet decontamination systems represent a significant logistical burden. Such systems may only be available to large-scale response teams and would be completely unavailable to working dogs in the field. Second, the selection of decontaminants and their appropriateness for all hazards removal needs to be directly identified.

Recent human decontamination studies published as part of the Primary Response Incident Scene Management (PRISM) guidelines have made recommendations that would remove more than 99% of chemical contamination: (1) move away from the hazardous area, (2) remove all clothes, (3) wipe skin with dry wipe (5, 6). If done quickly, their studies have shown that disrobing can reduce contamination by 90% and wiping the skin with a paper towel can further reduce contamination by an additional 9% (7). The testing performed to validate these guidelines shows that techniques other than the aforementioned “large quantities of soap and water” can be used to significantly remove chemical contamination from human skin. Further, the human skin decontamination techniques described by PRISM do not require a large logistical footprint and may be useful for removal of chemicals from working dogs at the point of exposure, despite the inability to remove clothing (other than leash, collar, tactical gear) or their fur.

In adapting canine-relevant PRISM guidelines, we suggest that the handler and working dog must quickly move away from the hazardous area. Handlers should decontaminate themselves first. Then, proceed to caring for their dog by removing all equipment from the dog (e.g., collar, vest, booties, goggles). The dog’s protective fur functions similar to human clothing, and unwanted substances should immediately be removed to prevent the compounds from wicking through the haircoat and onto the skin. This study evaluated the ability to prevent the transfer of toxic chemicals from fur to skin and remove CWAs utilizing a Canine Field Decontamination Kit (TDA Research, Golden, CO) that uses a series of three wipes (five dry, five wet, five dry) to: (1) initially remove gross contaminants from the fur with dry wipes, (2) wet the fur using surfactant wipes to clean away residual contaminants on the fur, and (3) remove final contamination and residual surfactant with dry wipes. The methods used in this study are designed to keep contaminants off and away from the canine’s skin, remove contaminants from the canine’s fur and skin, and prevent the transfer of hazardous chemicals or materials. In this context, we report on the laboratory evaluation of the waterless, nonreactive wipe decontamination procedure in comparison to the soap and water wash decontamination procedure to remove highly toxic CWAs from canine skin and fur.

## 2 Materials and methods

**Figure 2:**
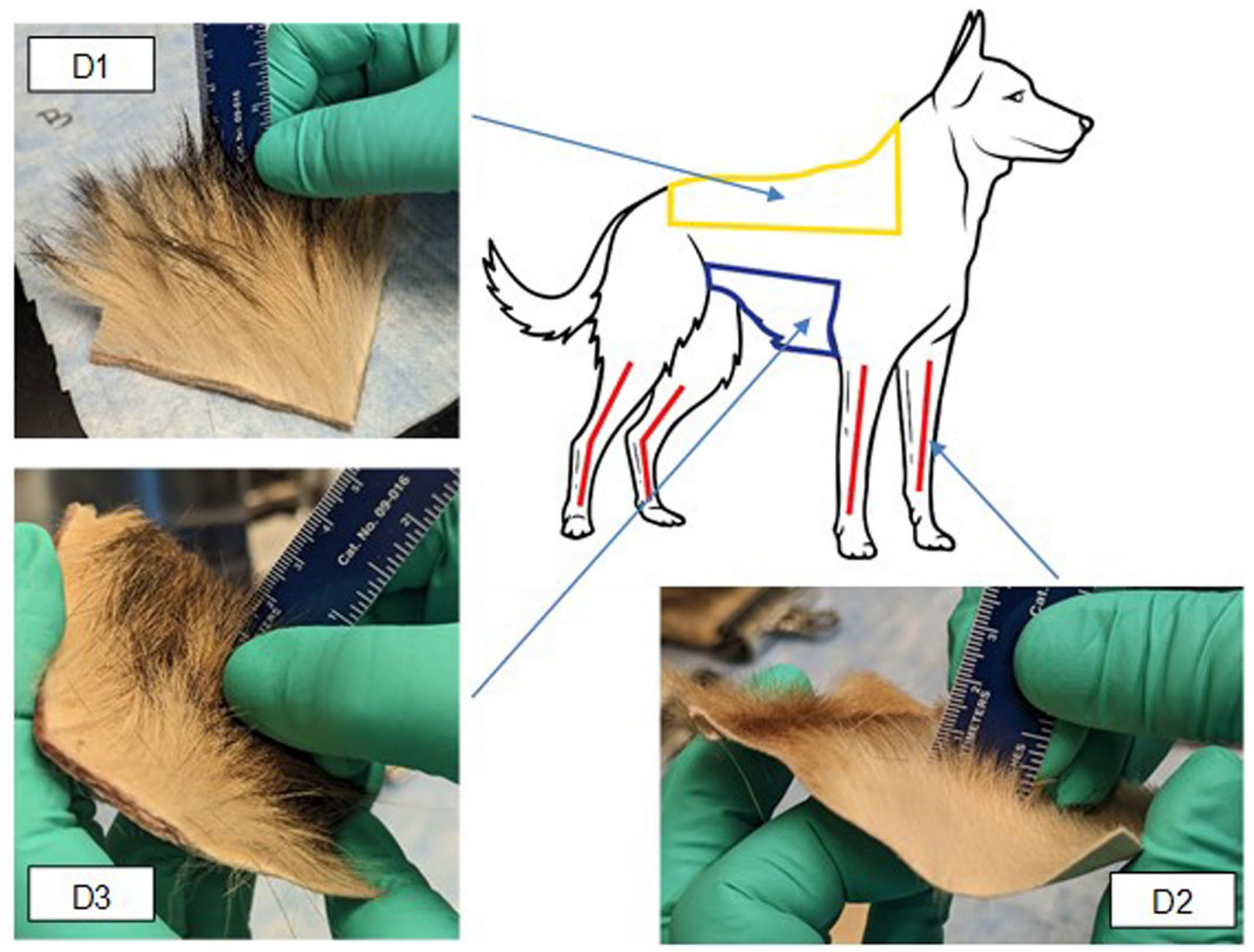
Anatomical location and example photos of collected canine tissue samples. Yellow area = D1 (dorsal axial), Red area = D2 (appendicular), blue area = D3 (ventral axial).

The CWAs sulfur mustard (HD; Chemical Abstracts Service (CAS) #505-60-2) and venomous agent X (VX; CAS #50782-69-9) synthesized by Battelle Memorial Institute (RRID: SCR_011112) were used for all testing. Purity samples of each CWA were generated by dissolving a known mass of each chemical into a known volume of acetone. Samples were analyzed on a gas chromatograph with a flame ionization detector and purity was determined by peak area. Acetone solvent blanks were used to correct for possible solvent contaminants. Purity was determined to be 99.9% for HD and 92.15% for VX. During testing, CWA was applied to the tissue samples with a positive displacement pipette. A commercially available Canine Field Decontamination Kit was evaluated during this study. The waterless, nonreactive decontamination kits were manufactured by TDA Research (Golden, CO) and obtained from Mantel Technologies (Fort Collins, CO). Canine cadaver tissue samples (intact skin/fur) were obtained from one canine in accordance with an approved animal welfare tissue use agreement. The canine was a one- to two-year-old male Belgian Malinois-German Shepherd mixed breed. Tissue samples were obtained from three anatomical locations on the canine and designated as Dog 1 (D1; dorsal axial), Dog 2 (D2; appendicular), and Dog 3 (D3; ventral axial). The samples from each anatomical location were freeze-dried and vacuum sealed. Upon receipt at the laboratory testing facility, all tissue samples were stored in a freezer at −20 ± 10 °C until they were needed for testing. Samples were allowed to thaw for at least 30 min prior to testing. Samples were “fluffed” with a non-lint towel to lift any fur that got matted post vacuum seal. For the testing of decontamination wipes, tissue samples were cut to approximately a 7.62 × 7.62-cm square to accommodate the manual dexterity required for wipe decontamination. The tissue samples used to test soap and water decontamination were 2.54 × 2.54 cm because the test procedure was conducted with a small brush and controlled application of a stream of water. Non-decontamination control samples gave similar results for both sized samples despite a higher probability of CWA penetration in the larger samples which received more droplets per sample. The anatomical locations of the collected tissue samples are shown in Figure 2. The fur length of each sample used for testing was measured to the nearest 0.5 cm with a ruler (Supplementary Table S1). Across all 7.62 × 7.62-cm and 2.54 × 2.54-cm samples, the average length for D1 was 2.7 cm [40% Relative Standard Deviation (RSD)], for D2 the average length was 1.0 cm (37% RSD), and for D3 the average length was 2.1 cm (21% RSD). As indicated by the fairly high RSD values, fur length varied across samples within a location. Fur length was measured for informational purposes by the testing lab, but tissue samples were provided for testing, precut and blind to the testing process. Thus samples were provided for each decontamination method at random with samples prepared from target areas on the canine, described above as D1, D2 and D3.

Testing of the Canine Field Decontamination Kit involved contaminating 7.62 × 7.62-cm (58 square centimeters (sq cm)) canine tissue samples with nine 2 μL droplets (18 μL total) of HD or VX. Testing of soap and water decontamination utilized 2.54 × 2.54-cm (2.54 sq. cm) canine tissue samples; therefore, the CWA contamination level was reduced by a factor of nine to two, 1 μL droplets (2 μL total) of HD or VX per sample. The non-decontamination control tests were performed first, then the wipe decontamination procedure evaluation, and finally the soap and water wash decontamination procedure testing. The same testing team at the Battelle laboratory oversaw the safety and security of all live chemical agent testing procedures.

For the soap and water wash, the selected soap was Dawn^®^ Ultra Dishwashing Liquid (Original Scent; Procter & Gamble). The soap was used as received (i.e., not diluted before use). The median amount of soap used to remove an oil-based contaminant in a previous study was 120 mL of Dawn^®^ (8). A 25 kg canine will have a surface area of approximately 0.864 square meters (9). Scaling 120 mL of soap based on the surface area of 8,638.7 sq. cm to the 2.54 sq. cm tissue sample indicated a 90 μL application of Dawn^®^ should be used for each sample. Also from previous studies, the average rinse water volume for canine decontamination is 39.8 L (10). Therefore, the volume of water for rinsing the 2.54 sq. cm samples was scaled down with a target of at least 29 mL allowed per sample. Room temperature distilled water was used for pre-wetting and rinsing soap off the tissue samples. Water was applied to the tissue samples in 10 mL increments using a calibrated peristaltic pump with a handheld applicator. For water rinsing, the tissue samples were placed on a stainless-steel screen at an angle above a waste collection container.

After performing decontamination of the HD and VX spiked canine tissue samples, the fur was trimmed away from the skin using electric trimmers. The fur was trimmed onto a clean, waxed-paper surface and then transferred to an empty jar. A small brush was used to remove any fur remaining on the hair trimmer. The skin was then cut into four pieces using stainless steel shears. Separately, the fur and skin samples were extracted in 250 mL glass jars with 100 mL of acetone. The jars containing fur and skin samples were placed in an ultrasonic bath and sonicated for 10 min and then allowed to sit for an additional 50 min. A calibrated positive displacement pipette was used to transfer 1.0 mL of solvent extracts to glass gas chromatography (GC) vials. Polytetrafluoroethylene (PTFE) syringe filters (1.0 μm pore size) were used to remove particulates. Sample extract concentrations of HD and VX for all test, control, and blank samples were calculated in units of μg/mL from GC/mass spectrometry (MS) analyses. Mass recovered from fur and skin samples via solvent extraction was determined according to Equation 1:


(1) $${\text{Mass}}_{\mathrm{Rec}}={\text{Conc}}_{\mathrm{Ext}}\times {\mathrm{Vol}}_{\mathrm{Ext}}$$

Where:

Mass_Rec_ = HD or VX mass recovered from test sample (μg).

Conc_Ext_ = test sample HD or VX extract concentration (μg/mL).

Vol_Ext_ = volume of extraction solvent (mL).

The total mass of recovered CWA was calculated by summing the mass recovered from the fur and skin samples. The method quantitation limit (MQL) was determined using Equation 2:


(2) $$\mathrm{MQL}=\mathrm{LOQ}\times {\mathrm{Vol}}_{\mathrm{Ext}}$$

Where:

MQL = method quantitation limit (μg).
LOQ = limit of quantitation (μg/mL).
Vol_Ext_ = volume of extraction solvent (mL).

Based on Equation 2 and a 100 mL solvent extraction volume, the MQL values for the 7.62 × 7.62-cm wipe samples were 0.1 μg/mL × 100 mL = 10 μg for both fur and skin samples. Percent reduction of CWA was calculated using Equation 3.


(3) $${\mathrm{Red}}_{\mathrm{CWA}}=\frac{{\text{Mass}}_{\text{Chal}}-{\text{Mass}}_{\mathrm{Rec}}}{{\text{Mass}}_{\text{Chal}}}\times 100$$

Where:

Red_CWA_ = Percent CWA reduction.
Mass_Chal_ = Average total CWA challenge mass for process controls (μg).
Mass_Rec_ = Average total CWA mass recovered for test samples (μg).

### 2.1 Wipe decontamination procedure

Each approximately 7.62 × 7.62-cm tissue sample was contaminated with HD or VX as described above. To provide safe handling, tissue samples were held to 10.16 × 10.16-cm acrylic sheets using 7.62 cm wide steel clips. All materials used for sample handling were single-use and were decontaminated and disposed of at the end of the test. All tests were performed at ambient laboratory temperature and relative humidity (RH) that were monitored but not controlled. For HD testing the average temperature was 19.4 °C and the average RH was 49.9%. For VX testing the average temperature was 20.8 °C and the average RH was 37.8%. Several different control samples were included in the testing. One negative control consisted of a tissue sample not spiked with CWA but carried through the entire test procedure to evaluate for potential cross contamination. Process controls (PC) consisted of canine tissue samples spiked with CWA that were not decontaminated but were carried through the entire test procedure to compare against test samples to evaluate the efficacy of the 3-wipe Canine Field Decontamination Kit. Spike controls consisted of PTFE disks spiked at the same level as the test samples and immediately extracted in solvent.

The CWA contamination was allowed to dwell on the tissue sample for a period of 30 min. The samples were uncovered in a chemical fume hood during the dwell time. At the end of the dwell period, use of the Canine Field Decontamination Kit was performed in accordance with the kit use instructions. The wipe decontamination process utilized three microfiber wipes, starting with a dry 25.4 × 25.4-cm wipe, followed by a surfactant wet 25.4 × 25.4-cm wipe, and then another dry 25.4 × 25.4-cm wipe. The wet wipe surfactant is a proprietary blend that is not commercially available in any other form. Each separate wipe was used for approximately two minutes per sample. The wiping process for each tissue sample took approximately six minutes total. Each wipe was used only once and then disposed of. Only one Battelle staff member performed decontamination with the wipes, which reduced operational variability. The entire sample was wiped regardless of CWA spike location. Wiping movements included a pinch/pull technique and moving against the fur growth direction were used to prevent physical CWA transfer to the skin. Decontamination wipes were not analyzed for CWA, as nothing in the wipe was expected to neutralize or otherwise detoxify the CWAs. Following decontamination, fur and skin samples were analyzed separately and quantified (Section 2.3).

### 2.2 Soap and water wash decontamination procedure

**Figure 3:**
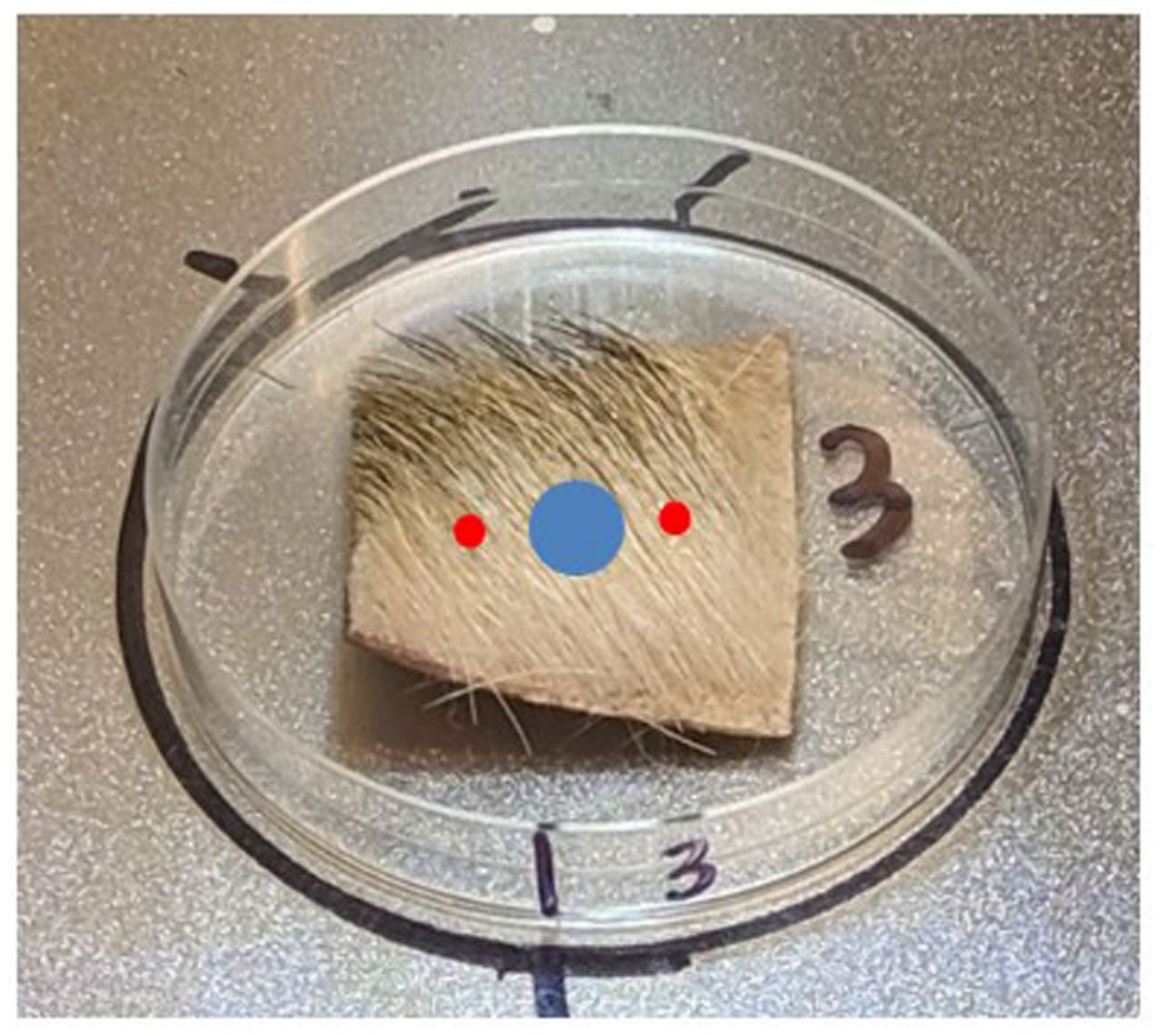
Notional placement of chemical warfare agent (red) and soap (blue).

Each approximately 2.54 × 2.54-cm tissue sample was contaminated using a 50 μL Class A gas-tight syringe fitted with a stepper to deliver two, 1 μL droplets of HD or VX near the center of each tissue sample (Figure 3). The CWA was then allowed to dwell on the tissue samples uncovered in a fume hood for 30 min.

Soap and water wash decontamination of the tissue samples followed four steps:

1. Rinse the tissue sample thoroughly with distilled water.
2. Work the soap into the fur. Ensure the soap reaches the skin.
3. Rinse with plain water.
4. Tap excess water off the tissue sample.

The tissue sample was held at an angle and 10 mL of water was applied across the surface of the tissue sample to rinse and pre-wet the sample (Step 1). A single 45 μL drop of Dawn^®^ dish soap was applied near the center of the tissue sample using a calibrated pipette. The soap was not placed on top of the CWA application locations (Figure 3). The soap was then worked into the fur using a toothbrush for approximately 30 s (Step 2). A new toothbrush was used for each sample. The sample was then held at an angle and water was applied across the sample to remove the soap (Step 3). Laboratory assessment of the 2.54 sq. cm ventral axial (D3) tissue samples indicated that 45 μL of Dawn^®^ provided sufficient soap while a minimum of 50 mL water rinse volume was needed to visibly remove soap from the samples. A 50 mL volume was also selected for the appendicular (D2) samples (which had shorter fur than D3) while a 70 mL water rinse volume was selected for the dorsal axial (D1) samples (which had longer fur). Each tissue sample was rotated as needed to ensure proper rinsing. After rinsing with 50 mL or 70 mL of water, a final 10 mL of water was used to remove any residual soap that may have been transferred to the underside (hypodermis) of the sample.

There was no wait time following working soap into the fur and rinsing with water. Rinse water was not analyzed for HD or VX. To simulate the canine shaking to remove water, the tissue samples were picked up with tweezers and gently tapped against the rinse screen to remove excess water (Step 4). The fur was not dried with a cloth as this may have aided in removal of any residual CWA. Each sample was allowed to air dry for 30 min, however, samples were still damp prior to fur removal. Fur and skin samples were analyzed separately and quantified (Section 2.3).

### 2.3 Fur removal and solvent extraction procedures

Electric trimmers were used to remove fur from each tissue sample directly into a clean, empty 100 mL glass jar. The skin sample was then placed in a separate 100 mL glass jar; 7.62 × 7.62-cm samples were cut to fit into the glass jar prior to extraction, the 2.54 × 2.54-cm skin samples did not need to be cut into smaller pieces prior to extraction. A 50 mL volume of acetone was added to each jar. CWA was quantified in the solvent extracts by GC/MS analysis. The MQL for soap and water evaluation was 5.0 μg for both fur and skin based on Equation 2.

## 3 Results

### 3.1 Chemical warfare agent interaction with fur

**Figure 4:**
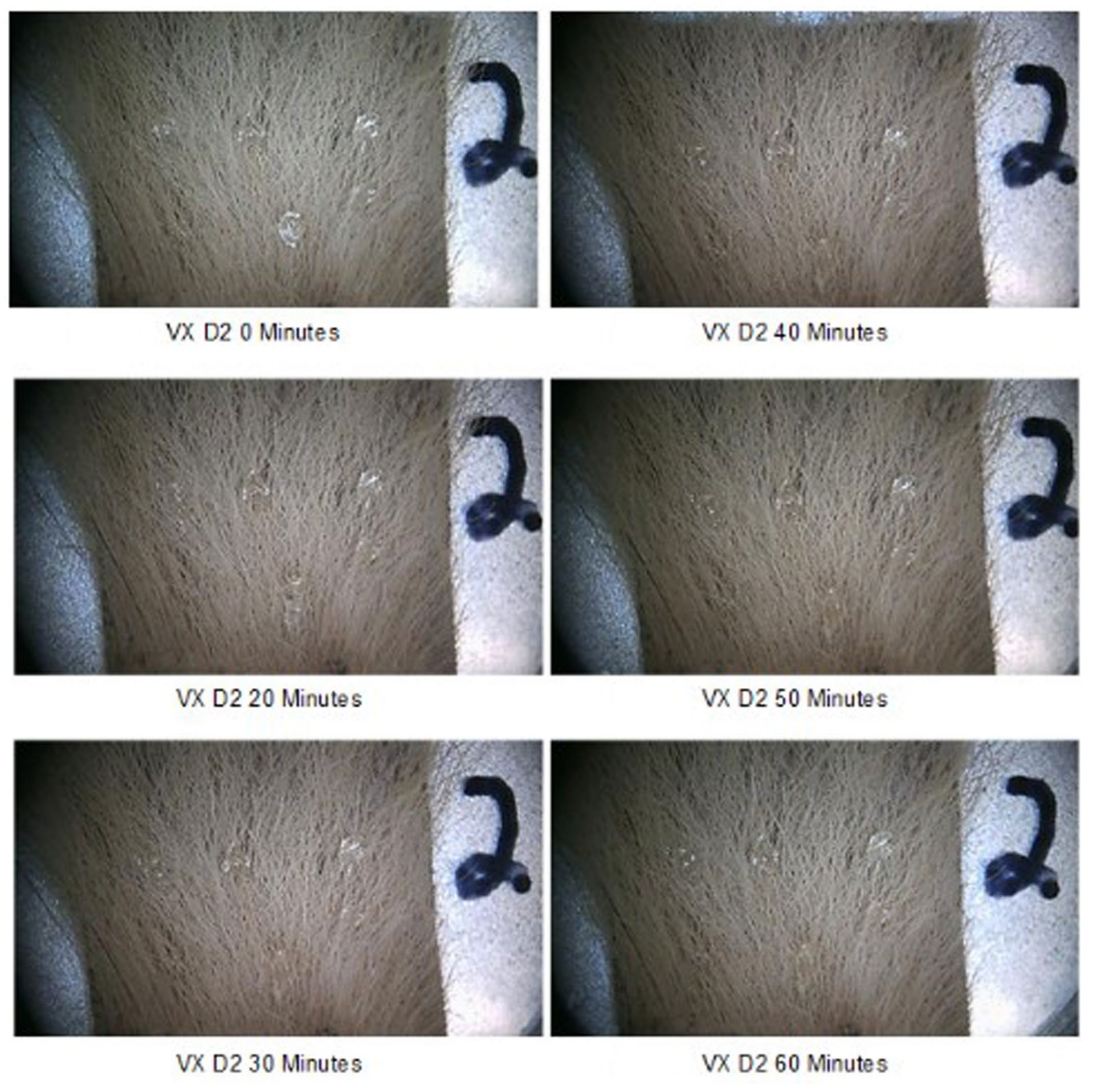
Six, 5 μL VX droplets on D2 appendicular tissue sample over time.

The behavior of two CWAs on the fur was visually evaluated and photographed over time to determine their interaction and movement on and in fur. Six 5 μL droplets HD or VX (30 μL total) were spiked onto samples of D1, D2, and D3 type fur samples and observed over 60 min. For the majority of samples, the droplets slightly spread along the length of the fur immediately after application. The spread was consistent with fur supporting the weight of the droplet. Some droplets beaded on the fur; this can be seen for VX on sample D2 (Figure 4). Both CWAs were evaluated, minimal movement of the CWA on the fur was observed for a duration of 60 min. Some additional spread of HD was observed on D1 and D3 fur samples after 10 min, with no changes after that timepoint. As an example of behavior over time, Figure 4 shows chronological photos of VX on D2 at each timepoint (T = 0 to T = 60 min). Two beads of VX on the D2 sample spread slightly after 10 min with no observable changes after that timepoint. Since no droplet movement following CWA application was observed between 30 and 60 min, a CWA dwell of 30 min was selected for testing.

### 3.2 Wipe decontamination procedure results

Three different types of controls (spike, process, and negative control) were prepared for each set of Canine Field Decontamination Kit tests. Based on CWA purity, density, and application of nine, 2 μL droplets on the 7.62 × 7.62-cm tissue samples, the nominal HD sample spike mass was 22,800 μg and the VX sample spike mass was 16,700 μg. Spike controls were prepared by applying CWA to inert PTFE disks in the same manner as the test samples, but were immediately extracted in solvent. Analysis of the spike control samples indicated that average HD application was 107% of nominal and average VX application was 103%, well within the expected spike control range of ±20% of nominal.

Tissue samples were used in the process controls to evaluate the total recovery of CWA from tissue samples that were not decontaminated, these samples also helped to establish the distribution of CWA between fur and skin. The process control results were also used as a baseline for comparison to the decontamination results. For each set of tests, one tissue sample from each anatomical location (D1, D2, and D3) was prepared as a process control. Following application to the fur, CWA was allowed to dwell for 30 min like the decontamination test samples. The fur was then removed from the skin, with the fur and skin extracted separately. On average, the total recovery of HD (from both the fur and skin) was 83.5% relative to the spike control average. The total process control VX recovery averaged 88.9% relative to the spike control average. The slightly lower CWA recovery for the process controls is likely related to minor CWA losses during sample processing.

HD control results are shown in Table 1. Most of the HD remained on the fur. For sample D2, more HD was transferred to the skin than the other two sample types, which may be related to D2 samples having the shortest fur length. Similarly, the majority of VX remained on the fur with the exception of sample D2, where about half of the VX was transferred to the skin, likely related to the short fur length which may have allowed VX droplets to contact the skin. VX control results are shown in Table 2. The VX D2 process control sample had a measured fur length of 0.5 cm, the shortest measured length for any individual D2 samples. Note that the D1skin sample was non-detect for VX, with a reported value less than the MQL of 10 μg. A conservative value of 10 μg was used for this sample in all calculations.

**Table 1:** HD wipe decontamination procedure controls on 7.62 × 7.62-cm canine tissue samples.

Control type	Sample ID	Mass of HD (μg) remaining on the fur	Mass of HD (μg) remaining on the skin	Total mass (μg)
Spike controls	Rep 1			24,400
	Rep 2			24,300
	Rep 3			24,800
	Average			24,500
Process controls	D1	19,200	75	19,300
	D2	20,000	410	20,400
	D3	21,600	81	21,700
	Average	20,300	189	20,500
Negative control	D3	< 10	< 10	< 20

**Table 2:** VX wipe decontamination procedure controls on 7.62 × 7.62-cm canine tissue samples.

Control type	Sample ID	Mass of VX (μg) remaining on the fur	Mass of VX (μg) remaining on the skin	Total mass (μg)
Spike controls	Rep 1			17,500
	Rep 2			16,800
	Rep 3			17,100
	Average			17,200
Process controls	D1	13,200	≤10	13,200
	D2	8,800	7,790	16,600
	D3	15,900	136	16,000
	Average	12,600	2,650	15,200
Negative control	D2	< 10	< 10	< 20

The results of the HD and VX wipe decontamination testing using the wipe decontamination procedure are shown in Tables 3, 4, respectively. Three replicates were tested for each skin location and each chemical agent type. For each replicate, the measured HD or VX remaining on the fur and skin as well as the summed total mass are provided. Average remaining mass for each sample type is also provided.

**Table 3:** Residual HD following wipe decontamination of canine tissue samples.

Sample ID	Replicate	Mass of HD (μg) remaining on the fur	Mass of HD (μg) remaining on the skin	Total mass (μg)	Average fur reduction vs. PC	Average total reduction vs. PC
D1	1	15	148	164		
	2	128	290	418		
	3	101	117	217		
	Average	81	185	266	99.6%	98.7%
D2	1	18	107	125		
	2	21	228	250		
	3	16	146	162		
	Average	18	160	179	99.9%	99.1%
D3	1	78	269	347		
	2	38	223	261		
	3	68	185	253		
	Average	61	226	287	99.7%	98.6%

**Table 4:** Residual VX following wipe decontamination of canine tissue samples.

Sample ID	Replicate	Mass of VX (μg) remaining on the fur	Mass of VX (μg) remaining on the skin	Total mass (μg)	Average fur reduction vs. PC	Average total reduction vs. PC
D1	1	23	59	82		
	2	470	16	486		
	3	290	53	344		
	Average	261	43	304	97.9%	98.0%
D2	1	33	75	108		
	2	47	489	536		
	3	37	27	64		
	Average	39	197	236	99.7%	98.5%
D3	1	291	162	453		
	2	26	39	65		
	3	391	63	453		
	Average	236	88	324	98.1%	97.9%

The average HD reduction on the fur was calculated by subtracting the average mass of HD remaining on the fur from the average total HD process control mass (20,500 μg) and dividing the difference by the average total process control mass. Recall that the process control average represents three different sample types, however, each type had similar results for HD on fur and for total measured HD, providing an acceptable baseline for comparison. For all replicates, less HD was detected on the fur than the skin, with the average fur reduction ranging from 99.6 to 99.9% compared to the process controls. The average total HD reduction using the Canine Field Decontamination Kit (compared to the process controls) was excellent for each sample type, ranging from 98.6 to 99.1%, with an average total reduction across all sample types of 98.8%.

The average VX reduction on the fur was calculated by subtracting the average mass of VX remaining on the fur from the average total VX process control mass (15,200 μg) and dividing the difference by the average total process control mass. Each process control had similar results for VX on fur and for total measured VX, providing an acceptable baseline for comparison. For each sample type, the average mass of VX remaining on the fur was higher than was observed for HD (i.e., less VX was removed from the fur relative to HD). However, the average VX fur reduction using the kit was still effective for each sample type, ranging from 97.9 to 99.7% compared to the process controls. The average total VX reduction using the Canine Field Decontamination Kit (compared to the process controls) was also excellent for each sample type, ranging from 97.9 to 98.5%, with an average total reduction across all sample types of 98.1%.

The average mass of HD transferred to the skin of the test samples was similar to the mass measured on the skin for the HD process controls, although the D2 process control was higher than the test samples. For the samples the Canine Field Decontamination Kit was used on, the mass of HD that may have been transferred to the skin related to wiping the fur was a small percentage (0.78 to 1.10%) of the average total HD process control mass (20,500 μg) as shown in Table 5. By comparison, the average amount of HD transferred to skin for the process control samples was 0.93%. This suggests minimal transfer of HD to the skin when following the Canine Field Decontamination Kit procedures.

**Table 5:** CWA transfer to skin during wipe decontamination of canine tissue samples.

Sample type	Mass of HD (μg) transfer to skin	HD transfer to skin vs. PC total	Mass of VX (μg) transfer to skin	VX transfer to skin vs. PC total
D1	185^a^	0.90%	43^a^	0.28%
D2	160^a^	0.78%	197^a^	1.30%
D3	226^a^	1.10%	88^a^	0.58%
D1 process control	75	0.36%	< 10	0.07%^b^
D2 process control	410	2.00%	7,789	51.2%
D3 process control	81	0.40%	136	0.89%

a Average value.

b MQL used for calculation. PC = process control.

The average mass of VX transferred to the skin of the D1 and D3 samples was similar to the D1 and D3 process controls, however, the D2 process control was about 40x higher than the average D2 sample type. As noted above, the extremely high VX mass on the D2 process control skin was likely related to the length of fur. For the test samples, the mass of VX that may have been transferred to the skin related to wiping the fur was a small percentage (0.28 to 1.30%) of the average total VX process control mass (15,200 μg) as shown in Table 5. By comparison, the average amount of VX transferred to the skin for process control samples D1 and D3 was 0.48%. This suggests minimal transfer of VX to the skin when following the Canine Field Decontamination Kit procedures.

### 3.3 Soap and water wash decontamination results

Soap and water wash decontamination testing was performed on smaller tissue samples, 2.54 × 2.54-cm, with CWA loading levels consistent with those used on the 7.62 × 7.62-cm wipe decontamination test samples. The three different types of controls (spike, process, and negative control) were prepared for each set of soap and water wash decontamination tests. Table 6 provides the results for the control samples from the HD soap and water wash testing and Table 7 provides the control sample results from the VX soap and water wash testing. Based on CWA purity, density, and application of two, 1 μL droplets the nominal HD sample spike mass was 2,540 μg and the VX sample spike mass was 1,920 μg. Spike controls were prepared by applying CWA to inert PTFE disks in the same manner as the test samples but were immediately extracted in solvent. Analysis of the spike control samples indicated that average HD application was 93.4% of nominal and average VX application was 99.5% of nominal, well within the expected spike control range of ±20% of nominal.

**Table 6:** HD soap and water decontamination procedure controls on 2.54 × 2.54-cm canine tissue samples.

Control type	Sample ID	Mass of HD (μg) remaining on the fur	Mass of HD (μg) remaining on the skin	Total mass (μg)
Spike controls	Rep 1			2,500
	Rep 2			2,490
	Rep 3			2,120
	Average			2,370
Process controls	D1	1,960	6.5	1,970
	D2	1,550	121	1,670
	D3	1,590	62	1,650
	Average	1,700	63	1,760
Negative control	D2	< 5.0	< 5.0	< 10

**Table 7:** VX soap and water decontamination procedure controls on 2.54 × 2.54-cm canine tissue samples.

Control type	Sample ID	Mass of VX (μg) remaining on the fur	Mass of VX (μg) remaining on the skin	Total mass (μg)
Spike controls	Rep 1			1,970
	Rep 2			1,910
	Rep 3			1,840
	Average			1,910
Process controls	D1	1,370	< 5.0	1,380
	D2	1,260	< 5.0	1,270
	D3	1,340	< 5.0	1,340
	Average	1,320	< 5.0	1,330
Negative control	D3	< 5.0	< 5.0	< 10

Process controls used tissue samples to evaluate the total recovery of CWA from tissue samples that were not decontaminated. This information is also helpful to determine the distribution of CWA between fur and skin. The process control results were used as a baseline for comparison to the decontamination results. For each set of tests, one tissue sample from each anatomical location (D1, D2, and D3) was prepared as a process control. Following application to the fur, CWA was allowed to dwell for 30 min. The fur was then removed from the skin, with the fur and skin extracted separately. On average, the total recovery of HD (from both the fur and skin) was 74.4% relative to the spike control average. The total process control VX recovery averaged 69.7% relative to the spike control average. The lower CWA recovery for the process controls is likely related to some CWA loss during sample processing.

The majority of the HD remained on the fur. For sample D2, more HD was transferred to the skin than the other two sample types (D1 and D3), which may be related to D2 samples having the shortest fur length. Note that all skin samples were non-detect for VX, with a reported value less than the MQL of 5.0 μg. A conservative value of 5.0 μg was used in all calculations. The lower contamination level for the VX D2 process control skin sample compared to what was measured for the ‘decontaminated’ VX D2 process control skin sample is likely related to the relatively longer fur length for this particular sample (1.0 cm) and lower VX spike volume; with only two, 1 μL droplets of VX applied there was less opportunity for VX to be transferred to the skin through the fur.

The results of the HD and VX soap and water decontamination testing are shown in Tables 8, 9, respectively. For each replicate, the measured HD or VX remaining on the fur and skin as well as the summed total mass are provided. Average remaining mass for each sample type is also provided.

**Table 8:** Residual HD following soap and water wash decontamination of tissue samples.

Sample ID	Replicate	Mass of HD (μg) remaining on the fur	Mass of HD (μg) remaining on the skin	Total mass (μg)	Average fur reduction vs. PC	Average total reduction vs. PC
D1	1	35	140	176		
	2	22	127	149		
	3	21	99	120		
	Average	26	122	148	98.5%	91.6%
D2	1	53	358	412		
	2	58	551	609		
	3	59	496	555		
	Average	57	468	525	96.6%	70.2%
D3	1	8.5	100	108		
	2	23	176	199		
	3	9.9	105	115		
	Average	14	127	141	99.2%	92.0%

**Table 9:** Residual VX following soap and water wash decontamination of tissue samples (*5.0 μg used to calculate average mass).

Sample ID	Replicate	Mass of VX (μg) remaining on the fur	Mass of VX (μg) remaining on the skin	Total mass (μg)	Average fur reduction vs. PC	Average total reduction vs. PC
D1	1	23	39	62		
	2	7.1	16	23		
	3	13	31	44		
	Average	14	29	43	98.9%	96.7%
D2	1	19	104	123		
	2	9.9	40	50		
	3	18	52	70		
	Average	16	65	81	98.8%	93.9%
D3	1	49	61	111		
	2^*^	< 5.0	23	28		
	3	6.0	21	27		
	Average	20	35	55	98.5%	95.8%

The average HD reduction on the fur was calculated by subtracting the average mass of HD remaining on the fur from the average HD total process control mass (1,760 μg) and then dividing the difference by the average total process control mass. Recall that the process control average represents three different sample types, however, each type had similar results for HD on fur and for total measured HD, providing an acceptable baseline for comparison. For all replicates, less HD was detected on the fur than the skin, with the average fur reduction ranging from 96.6 to 99.2%, compared to the process controls. The average total HD reduction using the soap and water wash procedure (compared to the process controls) was lower than observed for the tissue sample decontamination for each sample type, ranging from 70.2 to 92.0%, with an average total reduction across all sample types of 84.6%.

The average VX reduction on the fur was calculated by subtracting the average mass of VX remaining on the fur from the average total VX process control mass (1,330 μg) and dividing the difference by the average total process control mass. Each process control had similar results for VX on fur and for total measured VX, providing an acceptable baseline for comparison. For all replicates, less VX was detected on the fur than the skin, with the average VX fur reduction, ranging from 98.5 to 98.9% compared to the process controls. The average total VX reduction using the soap and water wash procedure (compared to the process controls) was slightly lower than observed for the canine sample decontamination, ranging from 93.9 to 96.7%, with an average total reduction across all sample types of 95.5%.

The average mass of HD transferred to the skin of the soap and water wash test samples was higher than the mass measured on the skin for the HD process controls. For the test samples, the mass of HD that may have been transferred to the skin by soap and water decontamination ranged from 6.90 to 27.00% of the average total HD process control mass (1,760 μg), as shown in Table 10. By comparison, the average amount of HD transferred to the skin for the process control samples was 3.6%. This indicates that the soap and water wash process led to the transfer of HD from the fur to the skin.

**Table 10:** CWA transfer to skin during soap and water decontamination of canine tissue samples.

Sample type	Mass of HD (μg) transfer to skin	HD transfer to skin vs. PC total	Mass of VX (μg) transfer to skin	VX transfer to skin vs. PC total
D1	122^*^	6.90%	29^*^	2.20%
D2	468^*^	27.00%	65^*^	4.90%
D3	127^*^	7.20%	35^*^	2.60%
D1 process control	6.5	0.37%	< 5.0	0.38%^a^
D2 process control	121	6.90%	< 5.0	0.38%^a^
D3 process control	62	3.50%	< 5.0	0.38%^a^

* Average value across all sample types.

a 5 μg used as the below limit of detection value.

The average mass of VX transferred to the skin of the soap and water wash test samples was also higher than the mass measured on the skin for the process controls. For the test samples, the mass of VX that may have been transferred to the skin related to soap and water decontamination ranged from 2.20 to 4.90% of the average total VX process control mass (1,330 μg), as shown in Table 10. By comparison, the average amount of VX transferred to the skin for the process control samples was 0.38%. This indicates that the soap and water wash process led to the transfer of VX from the fur to the skin. It is important to note that soap and water decontamination is a subject of research in mass casualty decontamination. Studies have shown that increased dermal absorption of toxic chemicals can occur during soap and water decontamination, known as the “wash in” effect (11).

## 4 Discussion

The evaluation of chemical warfare agents applied to canine fur indicate that HD and VX droplets of 2 μL and 5 μL spread along the hairs immediately after application. However, following the initial spread, neither HD nor VX appeared to substantially move further along the fur towards the skin over a 60-min period. Process control experiments performed during both decontamination procedure trials further showed that only small quantities of CWA were measured in the skin samples. These small quantities could be due to wicking and/or a combination of experimentally induced introduction of agent to the skin due to the fur trimming process and/or high air flow in the chemical fume hood exacerbating wicking from the fur to the skin over the 30-min dwell time. This demonstrates that CWAs do not quickly wick down the fur to the skin and do remain in the fur of the canine, further suggesting that canine fur offers some protection. However, with any CWA retention in the fur, the risk of contamination spread could be significant. Since these CWAs do not quickly wick down the canine fur into skin contact, the priority should be on handler safety. There may be time to evacuate contaminated areas and for the handler to don protective equipment before taking action to decontaminate their working dog.

The following observations were made during the waterless, nonreactive wipe decontamination testing (Canine Field Decontamination Kit):

- Total HD reduction was effective for all three canine tissue sample types, ranging from 98.6 to 99.1% reduction, with an average total reduction across all sample types of 98.8%.
- Total VX reduction was effective for all three canine tissue sample types, ranging from 97.9 to 98.5% reduction, with an average total reduction across all sample types of 98.1%.
- The mass of HD that may have been transferred to the skin by wiping the fur was a small percentage (0.78 to 1.10%) of the total applied HD process control mass. By comparison, the average amount of HD transferred to skin for the (non-decontaminated) process controls was 0.92%.
- The mass of VX that may have been transferred to the skin by wiping the fur was a small percentage (0.28 to 1.30%) of the total applied VX process control mass. By comparison, the average amount of VX transferred to skin for (non-decontaminated) process control samples D1 and D3 was 0.48%.

The following observations were made during soap (Dawn^®^) and water wash decontamination testing:

- Total HD reduction for all three tissue sample types ranged from 70.2 to 93.0%, with an average total reduction across all sample types of 84.6%.
- Total VX reduction for all three tissue sample types ranged from 93.9 to 96.7%, with an average total reduction across all sample types of 95.5%.
- The mass of HD that may have been transferred to the skin by the soap and water decontamination procedure ranged from 6.90 to 27.00% of the total applied HD process control mass. The average amount of HD transferred to skin for the (non-decontaminated) process controls was 3.60%.
- The mass of VX that may have been transferred to the skin by the soap and water decontamination procedure ranged from 2.20 to 4.90% of the total applied VX process control mass. The average amount of VX transferred to skin for the (non-decontaminated) process controls was 0.38%.

These results show that the waterless wipe decontamination procedure performed as well as or better than the soap and water wash decontamination for removal of HD and VX from canine fur. Analysis of skin extraction results indicate that soap and water wash decontamination procedures may transfer more HD and VX from fur to skin than the waterless wipe decontamination procedure. The transfer of CWAs from fur to skin can lead to a greater toxicological exposure for a canine. A summary of the live CWA results is shown in Table 11.

**Table 11:** Summary of live chemical warfare agent decontamination testing results.

Observation	Wipe decon process	Soap and water wash
HD total reduction (agent removal)	98.6–99.1% Average 98.8%	70.2–93.0% Average 84.6%
VX total reduction (agent removal)	97.9–98.5% Average 98.1%	93.9–96.7% Average 95.5%
Average HD skin transfer non-decon process control (transfer to skin)	0.92%	3.6%
Average VX skin transfer non-decon process control (transfer to skin)	0.48%	0.38%
HD skin transfer after decon (transfer to skin)	0.78–1.1%	6.9–27%
VX skin transfer after decon (transfer to skin)	0.28–1.3%	2.2–4.9%

The following scenario illustrates the benefits that the waterless wipe decontamination method can provide when dealing with highly toxic materials. According to the National Research Council Committee on Toxicology, the percutaneous lethal dose 50 (LD50) of VX in rabbits, pigs, and guinea pigs is 0.02 mg/kg (12). Assuming similar canine toxicity, a VX LD50 exposure to a 44-pound (20 kg) working dog would be 0.4 mg. The VX LD50 for a human handler is 0.14 mg/kg (13). A lethal VX exposure to a 154-pound (70 kg) handler would be 9.8 mg. It is also important to remember the risk of toxic contaminant transfer from the canine fur to the handler.

Escalating this scenario, 100 times the VX LD50 for the working dog would total 40 mg. VX density is 1.008 mg/uL so 100 lethal doses is 39.7 uL of agent. In this study, the VX volume applied to the tissue samples was 2 uL/drop. This type of exposure could occur when the dog is walking through grass and rubbing against bushes, picking up approximately 20 small droplets of oily residue on the fur. Professional working dog teams may be outfitted with tactical or protective equipment, like harnesses or vests, booties for paw protection, and goggles for eye protection. The majority of CWA would be transferred to the fur on the outer sides of the working dog and the threat of transfer and spread becomes a great risk to the handler and others sharing transport vehicles with the dog/handler team and their gear.

This study showed the HD and VX CWAs did not immediately wick from the fur to the skin over 60 min, allowing time for the handler to don protective equipment for themselves and evacuate the contaminated area with the working dog. Once away from the contaminated area, the handler can remove and discard the canine’s contaminated equipment and utilize a dry decontamination method like the Canine Field Decontamination Kit. Some CWA may be left in the fur, but with proper decontamination wipe use, the amount that could be transferred from the fur to other persons and objects is significantly less than a non-decontaminated canine. Assuming approximately 0.5% skin transfer (as shown in the laboratory test results) leading to canine absorption during 100 times the canine VX LD50 contamination (40 mg), the working dog would have only been exposed to 0.2 mg of VX. This is less than a lethal dose for a 20 kg canine, suggesting the working dog could survive both the contamination and decontamination event in this scenario.

Applying the same 100 times the canine VX LD50 contamination (40 mg) scenario and dog/handler team parameters to the soap and water wash decontamination method. The first wash may remove 93.9% of the agent, reducing the VX load from 40 mg to 2.44 mg. This is good from the standpoint that this is below the lethal dose for the handler. However, of the initial 40 mg burden of VX, 3% (1.2 mg) of the agent could transfer to the canine skin. Unfortunately, with 0.5% skin transfer during the initial contamination event and a further 3% skin transfer during the subsequent soap and water wash, the wash decontamination results in three times the canine VX LD50, and it is not likely for a dog to survive that level of exposure.

HD decontamination performance is similar to the described VX decontamination scenario, except the amount of HD transferred to the skin during a soap and water wash was much higher on average than VX (Table 11). While HD is slightly less toxic than VX (greater LD50), it is a vesicant. After a soap and water wash, significant burns and blisters would likely develop on the working dog’s skin over the subsequent 24 h. These wounds can be significant and would likely be very challenging for a working dog to survive. In addition to the removal of CWA, these examples illustrate the importance of decontamination method selection when removing toxic materials from a working dog.

Another advantage of the waterless wipe decontamination method is that the contaminants removed from the canine’s fur are contained on the wipes. The contaminated wipes can then be disposed of as a solid waste whereas the soap and water wash decontamination method creates a significant logistical challenge to dispose of up to 40 L of CWA contaminated water per wash (10). Finally, while performance comparisons between the traditional soap and water wash and the waterless wipe decontamination methods have been made, only the Canine Field Decontamination Kit procedure can be used in an austere environment without additional logistical support. The ability to conduct decontamination with wipes immediately after contamination and prior to further transportation for evacuation or medical care will significantly improve canine survival and prevent the transfer of contaminants to equipment, vehicles or personnel.

The authors believe that this laboratory study provides important information concerning techniques to remove toxic materials from working dogs. However, there are several limitations to the study that veterinarians and handlers should consider. This laboratory study used cadaver tissue samples from a single working dog (Belgian Malinois-German Shepherd mixed breed). The appendicular (D2) tissue samples had shorter fur length on average and increased CWA transfer to the skin. Additional testing of the effect of haircoat length is recommended and all personnel should exercise caution when extrapolating the results of this study to different canine breeds. This laboratory study was performed under controlled conditions and the canine tissues were freeze-dried to preserve them for testing, evaluated at room temperature, and were not moved during testing. Agent adsorption comparisons on living tissues were not performed during these tests; however, at elevated temperatures and with blood flow in the tissues, we hypothesize that HD and VX skin adsorption would likely increase in a real-world canine decontamination event. We believe this makes the study more relevant, highlighting that proper decontamination techniques must be used to prevent skin contact and thus adsorption of these dermal toxins. While the hypothetical decontamination performance scenario discussed in Section 4 could not include every real-world decontamination event variable, it does highlight the importance of successfully removing the dermal toxin and preventing it from reaching the canine’s skin. Laboratory testing showed that CWA droplets in the fur did not move on our laboratory samples. However, canine movement could influence agent movement. Shaking, self-grooming (licking), rolling, rubbing, scratching and other normal canine behaviors contribute to the spread of contamination and could pose additional risk to the canine, handler, veterinary medical provider and other personnel. This highlights the importance of enabling decontamination at the point of contamination with a wipe decontamination method. The handler or veterinary medical provider should don appropriate personal protective equipment and then address canine contamination before it is transferred from the fur. Prolonged exposure pending medical, hazardous materials team or other support is unlikely to be beneficial. This laboratory study did focus specifically on two chemical warfare agents. Not every chemical is expected to behave identically in a canine’s fur, however no other compound will be as toxic as these agents. It was the authors’ goal to evaluate two very toxic compounds to ensure the dry, waterless, non-reactive wipe decontamination method provided benefit against the most challenging toxins. Finally, due to the cost of toxic material laboratory testing time and canine cadaver tissue sample availability, the number of samples tested during this study was limited. Additional study samples would be needed to provide rigorous statistical scrutiny of each of these results. Despite these limitations, the results of this study are important to present to the working dog community of professionals for their consideration.

## 5 Conclusion

Special decontamination considerations must be made when dealing with the removal of hazardous, toxic, or reactive materials from working dogs. Canine fur provides a level of protection preventing direct skin contact with some chemicals. These chemicals can become trapped in the fur which could transfer to personnel, vehicles, and other equipment or animals. Methods that remove these chemical hazards without transferring the materials to the canine’s skin must be used so that the working dog or other personnel are not poisoned. Decontamination methods that provide a conduit from the fur to the skin must be avoided. Soap and water wash has been a traditional approach to decontaminate animals. This approach works when the contaminant is not toxic or is present in small enough quantities that it will not injure the animal. When highly toxic or large quantities of hazardous materials are present, techniques such as waterless wipe decontamination that prevent the transfer to the canine’s skin are required. The waterless wipe decontamination method and deployable kit described in this report has the advantage of removing toxins, so they are not transferred to the working dog’s skin or spread to the handler, transport vehicle, or other surfaces. The toxins are transferred to the microfiber wipes for containment and disposal. The wipes can be used at the point of contamination to reduce the time the working dog is directly exposed and that the handler is indirectly exposed. The wipes are small and lightweight and do not require additional logistical support in the form of water or spray systems. Based on the results of this study, we recommend decontamination of animals be accomplished by removing a majority of the contaminant using dry, waterless wipe methods prior to further handler contact, vehicle transport, or supplemental medical care which may include soap and water wash.

## Data Availability

The raw data supporting the conclusions of this article will be made available by the authors, without undue reservation.

## Footnotes

^1^This manuscript uses “military working dog” as the encompassing term for MWDs, multi-purpose canines and other categories of working dogs employed by DOD components and interagency partners.
