# Supplementary material for: Canine decontamination, a laboratory study evaluating proper techniques to remove toxic materials from working dogs
Source: Front Vet Sci. 2025 Sep 26;12:1649673. doi: 10.3389/fvets.2025.1649673 (PMC12511782; doi:10.3389/fvets.2025.1649673)
Supplement: Supplementary file 1 [file Table_1.DOCX]

**Table S1**: Tissue sample fur length measurements
